# Cocaine-Induced Synaptic Redistribution of NMDARs in Striatal Neurons Alters NMDAR-Dependent Signal Transduction

**DOI:** 10.3389/fnins.2020.00698

**Published:** 2020-07-14

**Authors:** Ilse Delint-Ramirez, Amir Segev, Asha Pavuluri, David W. Self, Saïd Kourrich

**Affiliations:** ^1^Department of Psychiatry, University of Texas Southwestern Medical Center, Dallas, TX, United States; ^2^Département des Sciences Biologiques—CERMO-FC, Université du Québec à Montréal, Montreal, QC, Canada

**Keywords:** NMDA receptor, ERK signaling, cocaine, postsynaptic density, extrasynaptic

## Abstract

The consequence of repeated cocaine exposure and prolonged abstinence on glutamate receptor expression in the nucleus accumbens has been extensively studied. However, the early effects of cocaine on NMDAR signaling remain unknown. NMDAR signaling depends on the subunit composition, subcellular localization, and the interaction with proteins at the postsynaptic density (PSD), where NMDARs and other proteins form supercomplexes that are responsible for the signaling pathways activated by NMDAR-induced Ca^2+^ influx. Here, we investigated the effect of cocaine on NMDAR subunit composition and subcellular localization after both intraperitoneal non-contingent cocaine and response-contingent intravenous cocaine self-administration in mice. We found that repeated cocaine exposure, regardless of the route or contingency of drug administration, decreases NMDAR interactions with the PSD and synaptic lipid rafts in the accumbens shell and dorsal striatum. We provide evidence that cocaine triggers an early redistribution of NMDARs from synaptic to extrasynaptic sites, and that this adaptation has implications in the activation of downstream signaling pathways. Thus, consistent with a loss of NMDAR function, cocaine-induced ERK phosphorylation is attenuated. Because early NMDAR activity contributes to the initiation of lasting addiction-relevant neuroadaptations, these data may hold clues into cellular mechanisms responsible for the development of cocaine addiction.

## Introduction

Previous studies have demonstrated an important role for glutamate transmission within the nucleus accumbens in drug addiction, a critical structure involved in reward and motivation. In particular, exposure to psychostimulant drugs, including cocaine and amphetamine, leads to changes in glutamatergic synaptic transmission that last beyond the detoxification period and that contribute to shaping addiction-relevant behaviors (Scofield et al., 2016; Wolf, 2016). Much of the work in this area has focused on slowly developing changes in AMPAR-mediated currents after prolonged withdrawal (Wolf and Tseng, 2012). In contrast, only a few studies have shown that activity of NMDARs during cocaine administration is critically involved in the subsequent development of changes in AMPAR activity (Sun et al., 2008; Zweifel et al., 2008; Ferrario et al., 2010; Brown et al., 2011; Mameli et al., 2011). However, whether early cocaine experience alters NMDAR-driven intracellular signaling, a mechanism that plays a critical role in addiction processes, is unknown. In particular, NMDAR-mediated Ca^2+^ currents can activate calmodulin that triggers several signaling pathways involved in drug addiction, including CaMKII, MAPK, CREB, and PKA pathways (Licata et al., 2004; DiRocco et al., 2009; Sarantis et al., 2009; Besnard et al., 2011; Pascoli et al., 2011a). However, the temporospatial properties and magnitude of the Ca^2+^ rise, and signaling pathways engaged, are strongly controlled by NMDAR subunit composition and subcellular localization (Petralia et al., 2009).

Studies to date have provided mixed results regarding the effect of cocaine administration on NMDARs, including subunit composition, expression, and function (Churchill et al., 1999; Lu et al., 2003; Ghasemzadeh et al., 2009a, b; Yamamoto and Zahniser, 2012; Ortinski et al., 2013; Wang et al., 2018). These discrepancies may be caused by several factors, such as the route of administration (intraperitoneal, i.p. versus intravenous, i.v.), the mode of administration (contingent versus non-contingent) and withdrawal time when accumbens tissue is collected. Determining the effects of cocaine exposure on factors that regulate NMDAR expression and function is key to understanding the effects on intracellular signaling that contribute to the development and maintenance of neuroadaptations known to shape addiction-relevant behaviors. For instance, 24 h after repeated i.p. cocaine treatment, CAMKII phosphorylation is decreased (Boudreau et al., 2009), an effect that could be a result of decreased synaptic levels of functional NMDARs. In contrast, response-contingent cocaine self-administration increases both GluN2B/GluN1 total protein (Lu et al., 2003; Ortinski et al., 2013) and NMDAR function (Wang et al., 2018), a neuroadaptation that is expected to enhance CAMKII phosphorylation. Because CAMKII phosphorylation is downstream of GluN2B-containing NMDARs, these data suggest that the mode of cocaine administration may differentially alter the expression and/or subcellular localization of NMDARs, and differentially alter signaling pathways that are triggered upon NMDAR activation.

NMDARs localized in the PSD are structurally organized (and spatially restricted) in a large macromolecular signaling complex comprising scaffolding and adaptor proteins that physically link receptors to specific kinases, phosphatases, and other downstream signaling proteins (Scannevin and Huganir, 2000). Importantly, activation of synaptic NMDARs stimulates ERK activity, whereas activation of extrasynaptic NMDARs inhibits ERK (Ivanov et al., 2006; Mulholland et al., 2008; Xu et al., 2009; Sun et al., 2016) (reviewed in Hardingham and Bading, 2010; Papouin and Oliet, 2014). Furthermore, the interaction of NMDARs with lipid rafts at the PSD promotes contact with specific signaling proteins (Delint-Ramirez et al., 2010). Therefore, determining the effect of cocaine on early NMDAR adaptations, such as subunit composition and subcellular localization, will shed light onto key mechanisms that are involved in the initiation of lasting effects of cocaine on synaptic strength and thereby the development of cocaine addiction.

Here, we conducted a comparative analysis of the effect of cocaine on NMDAR subunit composition and subcellular localization after non-contingent i.p. cocaine versus response-contingent i.v. cocaine self-administration. We found that both chronic cocaine self-administration and non-contingent cocaine injections disrupt NMDAR interactions with the PSD in nucleus accumbens shell and dorsal striatum. We also found that both cocaine treatment paradigms decrease NMDAR levels in lipid rafts at the PSD. Consistent with our hypothesis, this adaptation blocks further cocaine-induced ERK phosphorylation. Altogether, we provide evidence that cocaine triggers an early redistribution of NMDARs from synaptic to perisynaptic sites, and that this adaptation has implications in the activation of downstream signaling pathways thought to initiate the development of enduring neuroadaptations contributing to addiction.

## Materials and Methods

### Animals

Male C57/BL6 mice (bred on site; 7–12 weeks of age) from The Jackson Laboratory (Maine) were used in all experiments. Mice were group housed (4 animals per cage) and maintained on a 12-h light/dark cycle (light on at 7:00). After intrajugular catheterization (for cocaine self-administration), animals were individually housed. Mice had free access to water and rodent chow (2016 Harlan Teklad Global Diet). Animals who went through intrajugular catheterization were given daily i.p. injections of analgesic (Ketaprofen at 1.6 mg/kg) for 3 days post-op. During this recovery period, animals were provided with moist chow. On each day after this period, animals were treated with a triple antibiotic ointment at the surgery incision sites. Animals who showed declining health (poor appetite, decreased ambulation, and lack of grooming) were placed in heated cages until improvement of these measures. If deterioration continued, animals were taken out of the experiment and euthanized. All experiments were conducted in accordance with guidelines established by the Institutional Animal Care and Use Committee (IACUC) at the University of Texas Southwestern Medical Center (UTSW) (#2016-101853).

### Surgery

Animals were anesthetized with 100 mg/kg ketamine/10 mg/kg xylazine (i.p.) before surgical implantation of a chronic indwelling intravenous catheter. The catheters consisted of Silastic tubing (0.02 in. i.d. 0.037 in. o.d.; Green Rubber, Woburn, MA, United States) treated with tridode-cylmethyl ammonium chloride (TDMAC) heparin (Polysciences Inc., Warrington, PA, United States). Each catheter was secured at the jugular vein with Mersilene surgical mesh (General Medical, New Haven, CT, United States) and passed subcutaneously to exit the back through a 22-G cannula (Plastics One, Roanoke, VA, United States) embedded in dental cement on a Mersilene surgical mesh base. After surgery, animals received a prophylactic injection of penicillin (200,000 IU/kg/mL, 0.2 mL, i.m.), and antibiotic ointment was applied daily to the exit wound. Catheters were flushed daily with 0.2 mL of heparinized (20 IU/mL) bacteriostatic saline containing gentamycin sulfate (0.33 mg/mL) and 5 mg/mL of enrofloxacin (Baytril) to prevent clotting and curb infection. Catheter patency was checked before commencement of self-administration and at its conclusion using methohexital sodium (5 mg/mL, 0.05 mL i.v.).

### Drug Treatment

**FIGURE 1:**
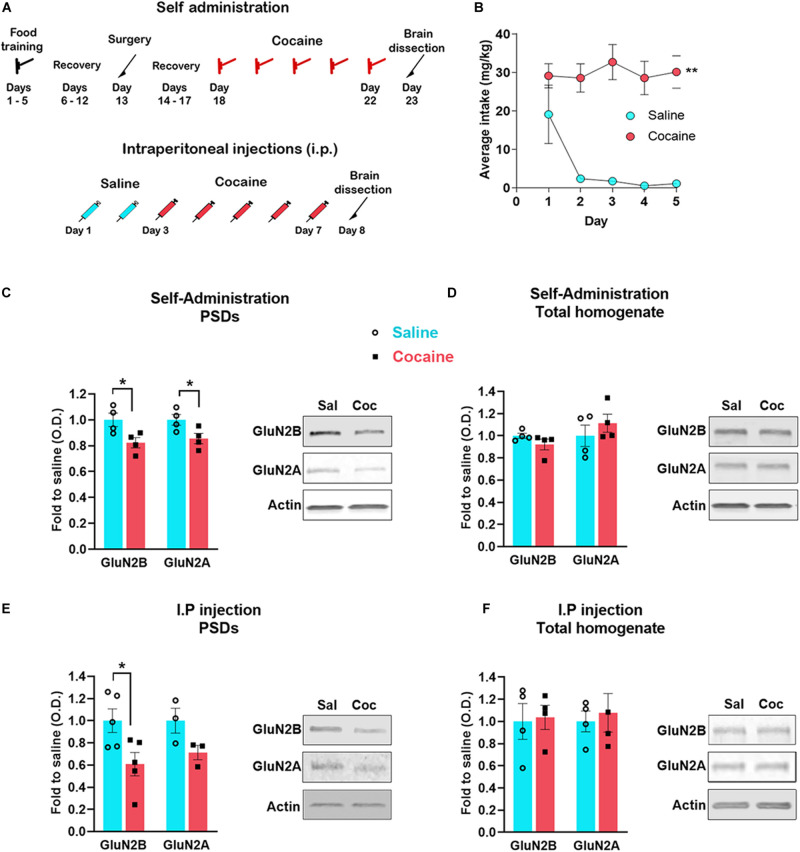
Timeline of the experiments **(A)**. Cocaine self-administration (mean number of cocaine intake during the 2-h sessions) **(B)**. Accumbens shell was dissected 24 h after the last self-administration session (*n* = 4 samples/group, 4 mice/sample, total of 16 animals/group) **(C,D)** or i. p daily injection of cocaine (*n* = 5 samples/group, 4 mice/sample, total of 20 animals/group) **(E,F)**. PSD enriched fraction was analyzed by western blot to detect the indicated proteins **(C,E)**; total protein extracted from the same samples was also analyzed **(D,F)**. Data are represented as mean ± SEM. *p < 0.05, **p < 0.01.

Prior to i.p. cocaine experiments, mice were injected (once daily) with saline for 2 days. Then, mice received 5 consecutive once-daily injections (Figure 1A) or 5 plus 1 challenge injection (Figure 4A) of saline and/or cocaine (15 mg/kg, i.p.; Sigma). Before i.v. cocaine experiments, mice received lever-press training for 25 mg sucrose pellets in operant test chambers with levers that retract at the end of each session (Med-Associates, St. Albans, VT, United States) on a fixed ratio 1 (FR1) reinforcement schedule in daily 1-h sessions. Lever-press training continued until an acquisition criterion of 30 sucrose pellets was consumed for 3 consecutive test days (mice were food-restricted for 16 h before each test session). For i.v. cocaine experiments, mice were trained to self-administer saline (0.05 mL) or cocaine (0.5 mg/kg/0.05 mL) for 5 days with 2-h-daily sessions on a FR1 schedule (Nugent et al., 2017). Mice were sacrificed either 24 h (short withdrawal) or 1 month (protracted withdrawal) after the last drug treatment, or 20 min after the challenge injection (no withdrawal). All treatments were performed between 10:00 and 14:00. The animals were allocated to different experimental groups using a complete randomization. The experimental group assignment was performed by a different person than the person who performed the biochemical analysis, that is, all protein assays and data analyses were performed blind to the treatments.

### Preparation of Synaptosomal Fractions, Synaptic Lipid Rafts, and PSDs

Total PSD, lipid rafts, and PSD without lipid rafts were isolated from the synaptosomal fraction as described earlier (Besshoh et al., 2005) with minor modifications (Delint-Ramirez et al., 2008, 2010, 2015; Morin et al., 2016; Sierra-Valdez et al., 2016). The synaptosomal preparation has been used to study proteins both at the presynaptic and at the postsynaptic membrane (Averna et al., 2015; Suresh and Dunaevsky, 2015). Synaptosomes contain the complete presynaptic terminal, along with the postsynaptic membrane, including perisynaptic compartments and the PSD (Corera et al., 2009). Synaptosomal fractions were prepared from the dissection of the accumbens shell or dorsal striatum using 4 animals per sample (except in Figure 4D). Indeed, when conducting biochemical assays requiring the fractionation technique, and especially when the brain region investigated is very small (here, the accumbens medial shell), we used at least three samples containing tissue pooled from 4 mice (i.e., at least 12 animals). This is consistent with previous studies using the fractionation technique (Bayes et al., 2011, 2012; Sierra-Valdez et al., 2016; Nuno et al., 2018). In order to obtain more homogenous tissue samples when harvesting the accumbens region, we restricted tissue collection to coronal dissections of the accumbens shell, and in particular, its rostro-medial subregion. This shell subregion is exclusively associated with appetitive behaviors (Reynolds and Berridge, 2002) and is most responsive to psychostimulant drugs (Ikemoto, 2010). To this end, mice were killed by cervical dislocation, brains were rapidly removed, then sliced and microdissected in ice-cold ACSF before homogenization in solution A (0.32 M sucrose, 0.5 mM CaCl_2_, 1 mM each of NaHCO_3_, MgCl_2_, and NaF, 2 mM sodium orthovanadate, 20 mM of glycerol 2-phosphate, complete protease inhibitors cocktail; Roche, Cat. no. 4693132001). Homogenization was performed by 12 up-and-down strokes with a Teflon glass homogenizer. The sample was centrifuged at 1000 × *g* for 5 min, the pellet was washed with 0.5 mL of solution A, and centrifuged again at 1000 × *g* for 5 min. The supernatants were mixed, and total extracts were separated in this step; the rest of the sample was centrifuged at 16,000 × *g* for 15 min to isolate the crude synaptosomal fraction. To isolate the total PSD, this last pellet was resuspendend in solution C (0.5 mL of 150 mM NaCl, 25 mM Tris–Cl buffer, pH 7.5, containing 50 mM NaF, 10 mM NaP_2_O_7_, 1 mM sodium orthovanadate, complete protease inhibitors cocktail, and 0.5% Triton X-100]. Triton X-100 extracts were incubated for 30 min at 4°C and centrifuged (16,000 × *g*, 4°C, 10 min) to isolate the detergent-insoluble pellet, that is, total PSD. To isolate synaptic lipid rafts, the crude synaptosomal fraction was resuspended in 0.5 mL of lysis buffer mixed with 2 M sucrose (1 mL), overlaid with 1 M (2 mL) and 0.2 M (1.5 mL) sucrose, and centrifuged for 18 h at 45,000 rpm (SW 60 Ti, 200,000 × *g*; Beckman) at 4°C. After centrifugation, the lipid rafts fraction was collected in the 1 M–0.2 M interface and the PSD without rafts was collected in the pellet.

To obtain a pure synaptosomal fraction, the resulting pellet (crude synaptosomal fraction) was suspended in solution B (solution A without CaCl_2_ and MgCl_2_), loaded on the top of a discontinuous sucrose gradient (equal volumes of 0.85, 1.0, and 1.2 M sucrose, each prepared in solution B) and centrifuged at 82,500 × *g* for 2 h. The band sedimenting between 1.0 and 1.2 M sucrose (synaptosomes) was collected. Synaptosomes were pelleted by centrifugation at 40,000 × *g* for 30 min in solution B.

### Western Blotting

Protein concentration was determined by Lowry assay. Equal protein concentrations from the fractions were subjected to sodium dodecyl sulfate–polyacrylamide gel electrophoresis (SDS-PAGE) and immunoblot for the indicated proteins. Primary antibodies were 1:1000 rabbit anti-GluN2A antibody (Millipore Cat. no. 07-632, RRID:AB_310837), rabbit anti-GluN1 (Millipore Cat. no. AB9864, RRID:AB_11212290), 1:10,000 mouse anti-GluN2B (Cat. no. 610416/7; BD Biosciences), 1:5000 mouse anti-actin (Bio-Rad Cat. no. MCA5775GA, RRID:AB_2571580), mouse anti-Phospho-p44/42 Erk (Tyr204)/(Tyr187) (D1H6G) (Cell Signaling Technology Cat. no. 5726, RRID:AB_2797617), rabbit anti-p44/42 MAPK (Erk1/2) (137F5) (Cell Signaling Technology Cat. no. 4695, RRID:AB_390779). Levels of immunoreactivity were quantified by densitometry using ImageJ 1.49 software (Wayne Rasband, National Institutes of Health, United States).

### Statistics

Data acquisition and analysis were performed blind to experimental conditions when possible. Results are presented as mean ± SEM overlaid with the scatter plot of individual data points. Statistical significance (using GraphPad Prism 8) was assessed using two-tailed Student’s *t* tests when data passed the Shapiro–Wilk normality test or two-way ANOVA and Tukey *post hoc* tests when appropriate normality of the data was reached. Mann–Whitney was used when data did not have a normal distribution or for experiments with *n* = 3 sample size. Tests for detecting outliers were not conducted, thus, data points were not excluded. No sample calculation was performed.

## Results

### Cocaine Self-Administration and Non-continent i.p. Cocaine Injections Both Decrease NMDAR Levels at the PSD in the Accumbens Shell

Using an electrophysiological approach in the accumbens shell, an earlier study provided indirect evidence that extrasynaptic NMDARs are increased 24 h after the last cocaine self-administration session or after direct stimulation of D1 receptors *in vitro* (Ortinski et al., 2013). These findings suggest that NMDARs redistribute from synaptic to extrasynaptic sites. To test this possibility, we assessed the interaction between NMDAR subunits and the PSD 24 h after both a sensitizing regimen of non-contingent cocaine and contingent cocaine self-administration. On average, mice self-administered 60 infusions during the 2-h sessions (i.e., 30 mg/kg per session) (Figure 1B). Here, we measured GluN2A and GluN2B specifically, which are responsible for the interaction of the NMDAR with the PSD (Delint-Ramirez et al., 2010). We found that 24 h after the last cocaine self-administration session (Figures 1A,B), mice exhibit decreased levels of GluN2B [*t*_(__6__)_ = 2.733, *p* = 0.034, *t* test] and GluN2A [*t*_(__6__)_ = 2.527, *p* = 0.045, *t* test] subunits at the PSD in the accumbens shell (rostro-medial subregion) (Figure 1C). The total amount of both subunits remain unchanged (GluN2B, *U* = 3, *p* = 0.2, Mann–Whitney; GluN2A, *t*_(__6__)_ = 1.449, *p* = 0.197, *t* test) (Figure 1D), and therefore, consistent with NMDAR subunit redistribution from synaptic to extrasynaptic sites. Similar to contingent cocaine self-administration, we found that non-contingent i.p. cocaine decreases GluN2B subunit levels at the PSD [*t*_(__8__)_ = 2.605, *p* = 0.013, *t* test] (Figure 1E) with no detectable change in total protein [*t*_(__6__)_ = 1.1884, *p* = 0.8567, *t* test] (Figure 1F). It is noteworthy to mention that GluN2A exhibits a statistically non-significant trend toward a decrease at the PSD (*U* = 0, *p* = 0.1, Mann–Whitney). Consistent with the obligatory role of GluN1 subunits for the proper assembly and surface delivery of NMDARs and the necessary role of GluN2 subunits for trafficking and distribution (Lau and Zukin, 2007; Paoletti et al., 2013), we also found a significant decrease in GluN1 subunits at the PSD after non-contingent i.p. cocaine [*t*_(__8__)_ = 3.916, *p* = 0.004, *t* test], and without changes in total protein levels [*t*_(__6__)_ = 0.9938, *p* = 0.3589, *t* test] (data not shown). These results indicate that repeated cocaine exposure, regardless of the route of administration and the contingency of drug exposure, decreases the interaction of the NMDAR with the PSD, suggesting reduced recruitment of NMDARs at the postsynaptic membrane.

### Cocaine i.v. Self-Administration or i.p. Injections Fail to Decrease the Amount of NMDARs in Synaptosomes

**FIGURE 2:**
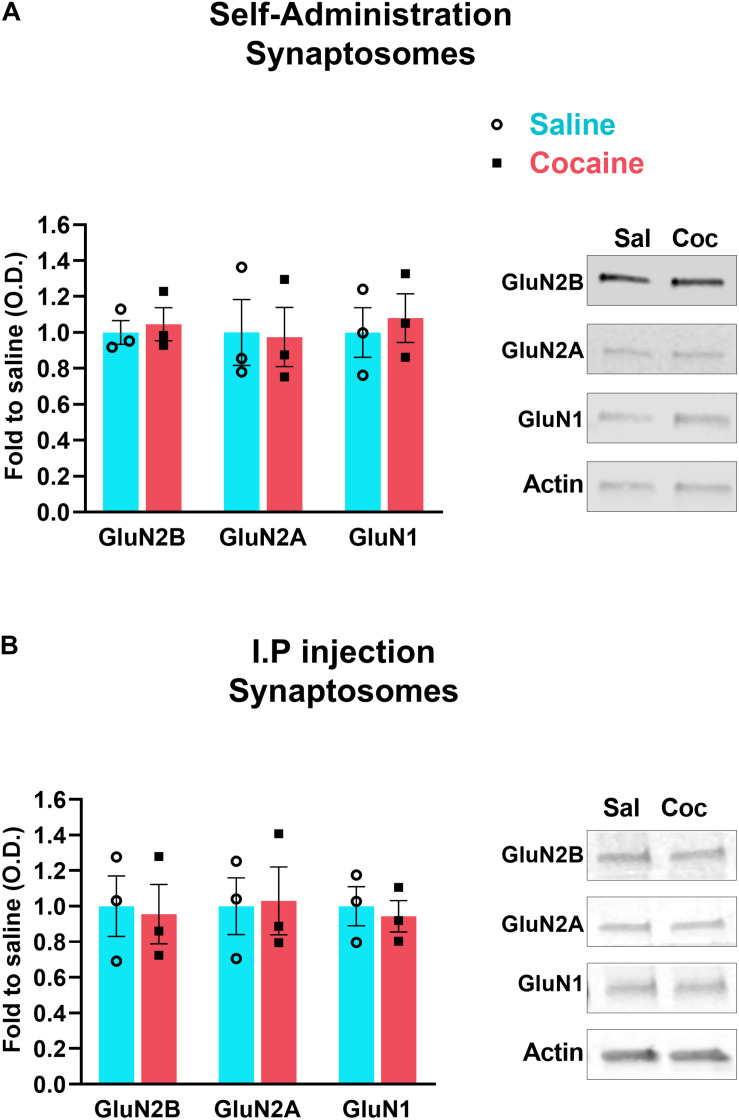
Accumbens shell was dissected 24 h after the last self-administration session **(A)** or i.p. daily injection of cocaine **(B)**. Synaptosomal fraction was isolated and analyzed by western blot to detect the indicated proteins. *N* = 3 samples/group, 4 mice/sample, total of 12 animals/group. Data are represented as mean ± SEM (minimum of 3 independent samples).

To determine whether cocaine-induced decrease in NMDAR subunits at the PSD is caused by decreased insertion of receptors into the synaptic membrane, we isolated synaptosomes from accumbens shell after both i.p. and i.v. cocaine paradigms. We found that neither non-contingent i.p. cocaine (GluN2B, *U* = 4, *p* > 0.9999, Mann–Whitney; GluN2A, *U* = 4, *p* > 0.9999, Mann–Whitney; GluN1, *U* = 4, *p* > 0.9999, Mann–Whitney) nor contingent cocaine self-administration (GluN2B, *U* = 3, *p* = 0.7, Mann–Whitney; GluN2A, *U* = 4, *p* > 0.9999, Mann–Whitney; GluN1, *U* = 3, *p* = 0.7, Mann–Whitney) decreases the density of NMDARs in the synaptosomal fraction (Figure 2). These data suggests that cocaine does not decrease membrane insertion of new receptors nor does it increase endocytosis, consistent with a redistribution of NMDARs within the synaptic membrane. Instead, combined with data in Figure 1, our data suggest that repeated cocaine induces a subcellular redistribution of NMDARs from the PSD to perisynaptic sites, thereby increasing the pool of extrasynaptic NMDARs.

### Cocaine Self-Administration Decreases the Interaction of NMDARs With the PSD and Lipid Rafts in the Dorsal Striatum

**FIGURE 3:**
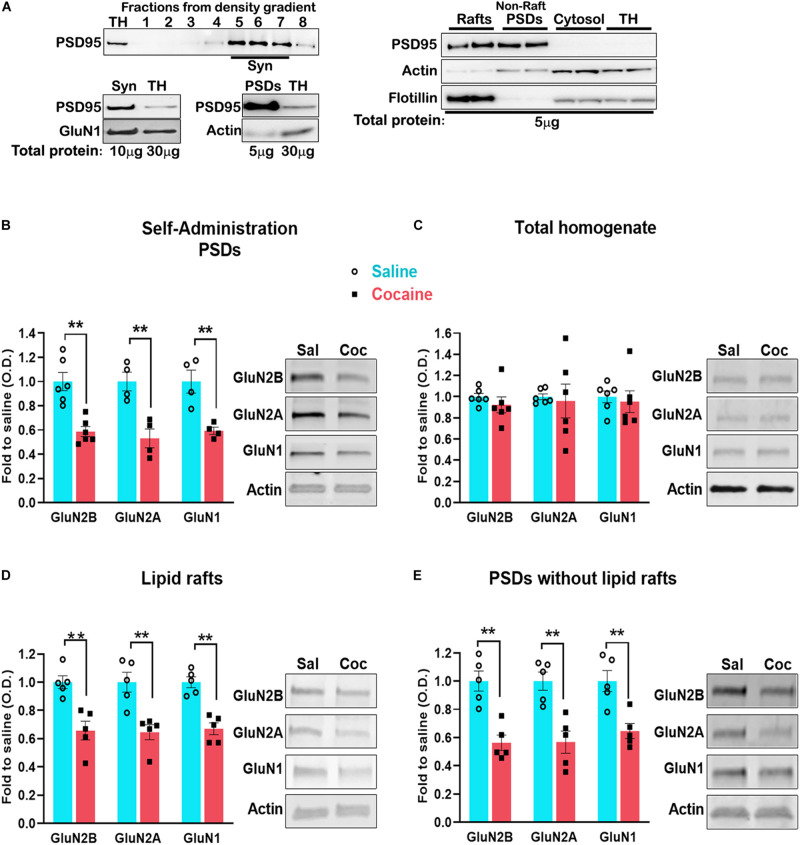
Blots of the cellular fractions obtained by density gradient (see section “Materials and Methods” for details) **(A)**. Dorsal striatum was dissected 24 h after the last self-administration session. Total PSD enriched fraction **(B)** was isolated and analyzed by western blot to detect the indicated proteins. Lipid raft **(D)** and PSD without rafts **(E)** were isolated from the total PSD and analyzed by western blot. Total protein **(C)**. For lipid rafts and PSD isolation, *n* = 6 samples/group, 4 mice/sample, total of 24 animals/group. Data are represented as mean ± SEM (minimum of 5 independent samples). **p < 0.01.

In order to verify the fractionation method, we show the distribution of the synaptosomal fraction (Syn) at the density gradient, using the PSD marker PSD-95 (Figure 3A *top left*); we also show the enrichment of PSD-95 in the synaptosomal fraction (10 μg of protein was loaded for the western blot) compared with the total homogenate (TH) (30 μg of proteins were loaded) (Figure 3A *bottom left*). The PSD fraction isolated from the synaptosomal fraction shows a higher enrichment of PSD-95. We also show the enrichment of PSD-95 both in the lipid rafts and the PSDs without lipid rafts fractions, and the presence of the lipid rafts marker Flotillin-1 in the lipid rafts fraction (Figure 3A *right*).

Lipid rafts are highly dynamic, sterol- and sphingolipid-enriched membrane domains specialized in signaling compartmentalization. They are present in dendrites and interact with the PSD (Suzuki et al., 2011). However, the association of NMDARs with lipid rafts is highly dynamic and changes during events associated with synaptic plasticity, such as spatial learning (Delint-Ramirez et al., 2008), after ischemic damage (Besshoh et al., 2005) and during development (Besshoh et al., 2007). Lipid rafts can be separated from the PSD by density gradient fractionation. However, pooling medial accumbens shell tissue from four mice did not yield a sufficient amount of tissue for lipid rafts isolation. To circumvent this technical barrier, we aimed to isolate lipid rafts from the dorsal striatum, a striatal region exhibiting similar NMDAR adaptations to that observed in the accumbens shell (Ortinski, 2014). First, we found that cocaine-induced decrease in NMDARs in the PSD fraction is recapitulated in the dorsal striatum (Figure 3B) (GluN2B, *t*_(__10__)_ = 4.841, *p* = 0.0007; GluN2A, *t*_(__6__)_ = 4.291, *p* = 0.005; GluN1, *t*_(__6__)_ = 4.080, *p* = 0.0065, *t* test) without changes in total NMDARs levels (Figure 3C) (GluN2B, *t*_(__10__)_ = 0.9414, *p* = 0.3687; GluN2A, *t*_(__6__)_ = 0.2471, *p* = 0.8098; GluN1, *t*_(__10__)_ = 0.3928, *p* = 0.7027, *t* test). Next, we pooled dorsal striatum from four animals and isolated lipid rafts from the PSD. We found that cocaine decreases the interaction of NMDARs with lipid rafts (Figure 3D) (GluN2B, *t*_(__8__)_ = 4.856, *p* = 0.0013, *t* test; GluN2A, *U* = 0, *p* = 0.0079, Mann–Whitney; GluN1, *t*_(__8__)_ = 5.835, *p* = 0.0004, *t* test) to the same extent as in the PSD without lipid rafts (Figure 3E) (GluN2B, *t*_(__8__)_ = 4.209, *p* = 0.0030; GluN2A, *t*_(__8__)_ = 4.241, *p* = 0.0028; GluN1, *t*_(__8__)_ = 3.846, *p* = 0.0049, *t* test). These results indicate that cocaine-induced decrease in NMDAR subunits (GluN2A, GluN2B, GluN1) is not a result of specific exclusion of the receptor from lipid rafts.

### Decreased NMDAR-PSD Interactions Are Associated With a Decrease in ERK Signaling

**FIGURE 4:**
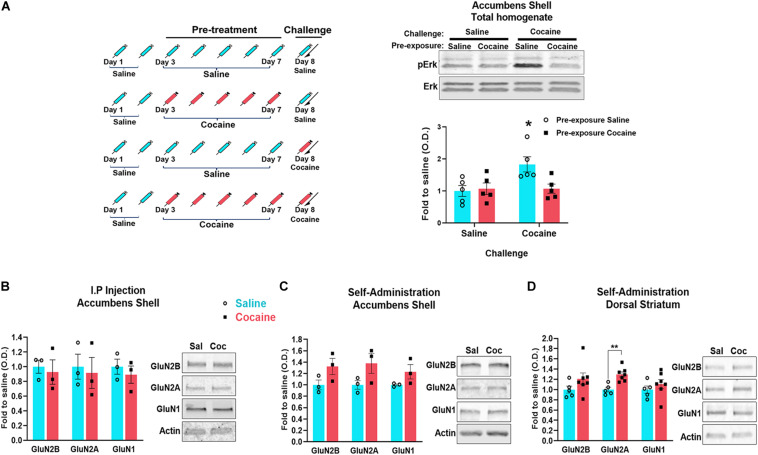
Timeline of the experiments: mice were i.p. injected 5 days with saline or cocaine (Pre-treatment) (**A** *left*). 24 h after the last injection, half of animals previously injected with cocaine were injected with an additional cocaine (challenge) and half with saline. Half of animals previously injected with saline were injected for the first time with cocaine (challenge) and half with an additional saline. 20 min after the last injection, the accumbens shell was dissected and the phosphorylation of ERK (pERK) was analyzed by western blot (1 animal/sample, *n* = 5 samples/group) (**A** *right*). Tissue was dissected 1 month after the last cocaine exposure. PSD enriched fraction was isolated and analyzed by western blot to detect the indicated proteins **(B–D)**. For accumbens shell **(B,C)**: *n* = 3 samples/group, 4 mice/sample. For dorsal striatum **(D)**: *n* = 5 samples/group, 1 mouse/sample (control group), and *n* = 7 samples/group, 1 mouse/sample (cocaine group). Data are represented as mean ± SEM. *p < 0.05, **p < 0.01.

It is well established that ERK signaling is activated by synaptic NMDARs and inhibited by non-synaptic NMDARs (Ivanov et al., 2006; Mulholland et al., 2008; Xu et al., 2009; Sun et al., 2016), and that a single cocaine administration increases ERK phosphorylation (pERK) (Pascoli et al., 2011b). Therefore, we used an ERK phosphorylation assay to provide additional evidence for cocaine-induced redistribution of NMDARs from synaptic to extrasynaptic sites. We compared pERK levels measured 20 min after a cocaine challenge (or saline as the control for the challenge) between animals that have received either saline or cocaine pre-treatment (5 days once-daily i.p. injections) (Figure 4B). We found that cocaine challenge increases pERK levels only in animals that received a saline pre-treatment (drug-naïve mice) (*F*_(__1_,_16__)_ = 5.026, *p* = 0.0395, two-way ANOVA, *post hoc* tests, *p* = 0.0458). Cocaine challenge did not enhance pERK in animals that were previously pre-treated with cocaine (*post hoc* tests, *p* = 0.9926) (Figure 4B). These results are consistent with reduced synaptic (and enhanced extrasynaptic) NMDARs after repeated cocaine administration.

### Repeated Cocaine-Induced Decrease in NMDAR–PSD Associations Is Not Lasting

Cocaine self-administration induces a long-lasting increase in AMPA/NMDA ratio in the accumbens (shell and core) that is associated with enhanced insertion of GluA2-lacking AMPARs, a neuroadaptation that contributes to enhanced drug seeking (Wolf and Tseng, 2012; Wolf, 2016). Because early cocaine-induced changes in NMDAR functions are thought to contribute to this slowly developing increase in AMPAR-mediated currents, we sought to determine whether cocaine-induced decrease of NMDARs at the PSD is also long lasting (Figure 1). Although we did not detect significant changes in NMDAR density at the PSD after 1-month withdrawal from non-contingent cocaine (GluN2B, *U* = 1, *p* = 0.2, Mann–Whitney; GluN2A, *U* = 1, *p* = 0.2, Mann–Whitney; GluN1, *U* = 0, *p* = 0.1, Mann–Whitney) (Figure 4B), we found that cocaine self-administration induces a statistically non-significant increase of GluN1 and GluN2B at the PSD in both accumbens shell (GluN1, *U* = 3, *p* = 0.7, Mann–Whitney; GluN2B, *U* = 4, *p* > 0.9999, Mann–Whitney) and dorsal striatum (GluN2B, *t*_(__10__)_ = 1.844, *p* = 0.1844, *t* tests; GluN1, *t*_(__10__)_ = 0.8792, *p* = 0.3999, *t* tests) (Figures 4C,D). It is noteworthy to mention that the low number of samples in Figure 4C may underlie statistically non-significant outcomes. However, to circumvent the need for pooling tissue from several animals, we conducted similar analyses with tissue from the dorsal striatum (Figure 4D: saline, *n* = 5; cocaine, *n* = 7), a larger brain region than the accumbens shell but that exhibits similar NMDAR adaptations (Ortinski, 2014). In these conditions, we found that levels of GluN2A are increased in the dorsal striatum [*t*_(__10__)_ = 4.172, *p* = 0.0019]. Together, this may reflect the formation of silent synapses and thereby the increase of NMDARs at the plasma membrane reported previously (Huang et al., 2009), and indicating that the loss of NMDARs from the PSD is not long lasting.

## Discussion

While each samples in experiments employing the fractionation technique contain tissue from several animals, we acknowledge that the low number of samples may have attenuated statistical power. In summary, our study shows that repeated contingent and non-contingent cocaine administration decreases the number of NMDARs at the PSD in both accumbens shell and dorsal striatum, whereas levels of NMDARs in the synaptosomal fraction or total protein remained unchanged. These results suggest that repeated daily cocaine triggers a redistribution of NMDARs from the PSD to the perisynaptic/extrasynaptic compartments. Corroborating these results, we found that cocaine-induced ERK phosphorylation in the accumbens shell, a mechanism that is dependent on synaptic but blocked by extrasynaptic NMDARs (Frasca et al., 2011; Bading, 2017), is blocked after repeated but not after a single cocaine administration. Therefore, these data suggest that repeated cocaine administration triggers an early mechanism that alters subsequent NMDAR-dependent signaling at the synapse.

### Dissociation of NMDARs From the PSD and Translocation to Extrasynaptic/Perisynaptic Regions

NMDARs are found both at synapses where they interact with PSD markers, and at extrasynaptic locations where they preferentially localize in perisynaptic areas (Perez-Otano and Ehlers, 2005; Zhang and Diamond, 2006), that is, near the border of the synaptic active zone (Petralia et al., 2010). Consistent with our results, cocaine self-administration and dopamine D1 receptor agonists decrease synaptic NMDAR-mediated currents and increase the contribution of extrasynaptic NMDARs signaling (Ortinski et al., 2013)—a mechanism that may be mediated by the decrease of NMDAR interactions with scaffold proteins. In particular, the C-terminal of NR2 subunits interacts with MAGUK family scaffold proteins at the PSD (e.g., PSD-95, PSD-93, SAP102, and SAP97) (Chen et al., 2015). Together, MAGUK proteins and NMDARs form signaling supercomplexes at the PSD through interactions with GKAP and Shank scaffolds (Cheng et al., 2003; Frank et al., 2004; Fernandez et al., 2009, 2017). Interestingly, repeated amphetamine administration, another psychostimulant drug that shares similar dopamine-elevating properties with cocaine, induces the ubiquitination and degradation of GKAP and Shank (Mao et al., 2009), suggesting that cocaine may destabilize NMDARs from the PSD through a similar mechanism.

### Cocaine Administration Decreases the Interaction of NMDARs With Lipid Rafts

Postsynaptic lipid rafts at the synaptic membrane interact with the PSD through several proteins, including the NMDAR (Renner et al., 2009; Suzuki et al., 2011). However, a substantial portion of synaptic proteins are specifically associated with either synaptic rafts or PSDs, supporting the notion that the two postsynaptic structures play different roles in synaptic functions (Becher et al., 2001; Ma et al., 2003; Guirland et al., 2004; Besshoh et al., 2005; Suzuki et al., 2011). For example, in lipid rafts, NMDARs interact with specific signaling proteins such as SRC family kinases and Arc, while NMDARs localized outside lipid rafts associate more closely with Syngap (Delint-Ramirez et al., 2010). The interaction of NMDARs with lipid rafts is highly dynamic upon stimuli or events that trigger synaptic plasticity, including spatial learning (Delint-Ramirez et al., 2008), ischemic treatment (Besshoh et al., 2005) and during development (Besshoh et al., 2007). Here, we show that repeated cocaine decreases the density of NMDARs at the PSD; therefore, we tested whether this decrease occurs specifically in the lipid rafts associated with the PSD or in the PSD fraction without rafts. Because accumbens shell does not yield enough tissue for lipid rafts analysis, we performed this analysis using dorsal striatum tissue, a brain region that recapitulates effects observed in accumbens shell (Figure 3). We found a similar decrease in NMDARs in both lipid rafts and non-rafts fractions, suggesting that decreased NMDAR density at the PSD affects signaling pathways at both lipid rafts and non-rafts subcellular compartments.

### Alteration of NMDAR Signaling

Transient activation of ERK pathway upon acute injection of cocaine is triggered by Ca^2+^ influx through NMDARs (Valjent et al., 2005; Girault et al., 2007). However, while the mechanism underlying extrasynaptic NMDAR-induced inhibition of pERK is still unknown, functional consequences of their activation have been extensively studied (Frasca et al., 2011; Bading, 2017). In particular and pertinent to the present study, disruption of ERK pathway via upstream pharmacological blockade of MEK prevents cocaine-induced expression of immediate early genes (IEGs) and the long-term behavioral effects of drug exposure (Valjent and Maldonado, 2000; Valjent et al., 2000; Choe and Wang, 2002; Choe et al., 2002; Salzmann et al., 2003). Consistent with these studies, reduced pERK after repeated cocaine (Figure 4) has been shown to correlate with decreased induction of IEGs observed after similar cocaine regimen (Belcher et al., 2009; Piechota et al., 2010; Echeverry-Alzate et al., 2012; Garcia-Cabrerizo and Garcia-Fuster, 2018). In sum, our study shows for the first time that while single cocaine i.p. exposure enhances pERK, repeated exposure to cocaine inhibits this signaling pathway through a mechanism resulting from the redistribution of NMDARs.

Although previous studies showed increased basal levels of pERK in the accumbens after chronic non-contingent cocaine administration (Wiegert and Bading, 2011), this increase occurred after 7 or 21 days of withdrawal and correlated with increased NMDARs at the PSD. Consistent with our results, this study found no change in basal levels of pERK or total NMDARs after 1-day withdrawal. In contrast, a previous study showed that pERK induction in the accumbens shell is not impaired when measured immediately after a 4-h session (long access) of cocaine self-administration in chronically trained rats (Edwards et al., 2007). While the experimental design used by Edwards et al. (2007) is fundamentally different than the one used here (pERK assessment 20 min after a single cocaine i.p. administration), these discrepancies might indicate that a long cocaine access triggers a slowly developing mechanism that potentiates pERK. This delayed mechanism could be involved in distinct signaling pathways than the mechanism involved in the transcription of IEGs. Importantly, cocaine-induced decreased levels of NMDARs at the PSD renormalizes during the withdrawal period and is no longer present when tested 1 month after the last cocaine self-administration session, indicating that the interaction of NMDARs with the PSD, and thereby pERK signaling, recovers during cocaine withdrawal. Indeed, although the number of samples is low (Figure 4C, *n* = 3), which may have prevented results from reaching statistical significance, we observe a trend for an increase of NMDARs at the PSD in the accumbens shell 30 days after cessation of i.v. cocaine exposure. However, it is noteworthy to mention that when conducting a similar analysis in dorsal striatum with more samples, we found that levels of GluN2A are increased (Figure 4D, *n* = 5–7).

In conclusion, chronic cocaine exposure independent of the route or mode of administration triggers a subcellular redistribution of NMDARs from the PSD to perisynaptic regions, thereby altering functional consequences of future cocaine exposure. This adaptation is important as the initial exposure to cocaine induces pERK, and thus, the transcription of IEGs—a mechanism that is thought to contribute to the development of stimulant addiction. We speculate that migration of NMDARs to perisynaptic membranes after repeated cocaine exposure may be a protective mechanism limiting subsequent induction of IEGs, and thus, the development of addiction processes. Nonetheless, further research is warranted to determine whether this adaptation is protective or further contributes to the perpetuation of stimulant addiction.

## Data Availability Statement

The raw data supporting the conclusions of this article will be made available by the authors, without undue reservation.

## Ethics Statement

The animal study was reviewed and approved by Animal Care and Use Committee at the University of Texas Southwestern Medical Center.

## Author Contributions

ID-R and SK: conceptualization, writing—original draft, visualization, funding acquisition, and supervision. ID-R: methodology and formal analysis. ID-R, AS, AP, and SK: investigation. ID-R, AS, DS, and SK: writing—review and editing. All authors contributed to the article and approved the submitted version.

## Conflict of Interest

The authors declare that the research was conducted in the absence of any commercial or financial relationships that could be construed as a potential conflict of interest.

## Abbreviations

AMPA: α-Amino -3-hydroxy-5-methyl-4-isoxazolepropionic acid
Arc: activity-regulated cytoskeleton-associated protein
CaMKII: calmodulin-dependent protein kinase II
CREB: cAMP response element-binding protein
ERK: extracellular signal-regulated kinase
GKAP: guanylate-kinase-associated protein
MAGUK: membrane-associated guanylate kinases
MAPK: mitogen-activated protein kinase
MAPKs: mitogen-activated protein kinases
MEK: MAPK/ERK kinase
NMDA: N-methyl -D-aspartate
PKA: protein kinase A
SAP: synapse-associated protein.
